# A rare case of Castleman disease presented with diffuse ground glass nodules in both lungs: Case report

**DOI:** 10.1097/MD.0000000000040681

**Published:** 2025-04-04

**Authors:** Xiaotong Guo, Caixia Zhu, Fen Zhang, Juan Chen, Kedong Zhang

**Affiliations:** a Department of Respiratory and Critical Care Medicine, The General Hospital of Ningxia Medical University, Yinchuan, China; b Department of Rheumatology, The General Hospital of Ningxia Medical University, Yinchuan, China.

**Keywords:** castleman disease, diffuse ground glass nodules, multicentric CD, unicentric CD

## Abstract

**Rationale:**

Castleman disease (CD) is a rare chronic lymphoproliferative disorder with unclear etiology and pathogenesis. It is divided into unicentric CD, which involved a single enlarged lymph node or region of lymph nodes, and multicentric CD, which involved multiple lymph node stations. Chest computed tomography (CT) scan is of great value in the diagnosis and differential diagnosis of the disease. CT scan mainly present large soft tissue mass in lungs and multiple mediastinal lymph node enlargement. Multiple ground glass nodules in both lungs are rare in CD patient.

**Patient concerns:**

A 48-year-old woman presented with chest tightness, shortness of breath, cough, and sputum. The chest CT scan showed multiple ground glass nodules in both lungs.

**Diagnoses:**

Multicentric Castleman disease was diagnosed through biopsies of the mediastinal 4R group, 7th group lymph nodes and the right inguinal lymph nodes.

**Interventions:**

Initial treatment with prednisone was administered, but due to the absence of significant radiological improvement on chest CT after 1 month, a systemic chemotherapy was initiated.

**Outcomes:**

After 6 cycles of systemic chemotherapy with cyclophosphamide, vincristine, and prednisone, the patient chest CT and clinical symptoms improved. Currently, the patient is still receiving low-dose prednisone and cyclophosphamide orally for long-term maintenance treatment.

**Lessons:**

CD that presents multiple ground glass nodules in both lungs is rare. It is easily confused with other diseases, identified diagnosis is depend on pathological examination. The accuracy of clinical subtype and histopathogenic type are important for treatment and outcome.

## 1. Introduction

Castleman disease (CD) is a rare chronic lymphoproliferative disease, and was firstly reported by Castleman in 1956.^[1]^ Clinically, according to the distribution of swollen lymph nodes, CD is divided into unicentric CD and multicentric CD (MCD). The main manifestations of unicentric CD are single-site lymph node enlargement, lack of specific clinical manifestations and abnormal laboratory indicators. MCD involved multiple lymph node stations as well as a wide spectrum of clinical and laboratory abnormalities.^[2]^ The most common features of MCD are constitutional symptoms, fluid accumulation, cytopenias, and liver and kidney dysfunction. About 1/3 of MCD patients develop malignant tumors, such as Kaposi sarcoma and malignant lymphoma.^[3]^ CD that presents with diffuse parenchymal lung disease is extremely rare. Here, we report a 48-year-old woman diagnosed with MCD, which presents with diffuse nodules in both lungs.

## 2. Case presentation

A 48-year-old female was admitted to our department with a 3-week history of cough, expectoration and reduced exercise tolerance, no hemoptysis, fever, chest pain, and other symptoms. The patient had a history of anemia for several years, and was diagnosed as “iron deficiency anemia,” irregular oral administration of iron sucrose. She denied any history of hypertension, diabetes, heart disease, liver disease, or kidney disease. She denied any recent travel, exposure to any chemicals, or any pets. The mother of the patient died of blood system disease 1 year ago (details are not specified), and her father, 1 sister and 2 brothers are healthy.

Physical examination findings: vital signs demonstrated temperature of 36.5 °C, with a heart rate of 86 beats/min, a blood pressure measurement of 125/70 mm Hg, and a respiratory rate of 22 breaths/min with oxygen saturation of 91% on room air. The breath sounds in both lungs were thickened, and the moist rales could be heard in both lungs. No enlargement was found in the neck or bilateral axillary lymph nodes. Both sides of the groin area can reach about 1 × 1 cm enlarged lymph nodes, mobile, firm, and painless. No obvious abnormalities were observed in the rest of the physical examinations.

Arterial blood gas analysis while breathing ambient air showed an arterial oxygen tension of 59 mm Hg, carbon dioxide arterial tension of 46 mm Hg, pH of 7.39 and arterial oxygen saturation of 93%. Blood tests noted decreased hemoglobin (89 g/L, normal 120–160 g/L), increased C-reactive protein (72.9 mg/L, normal < 6 mg/L), increased erythrocyte sedimentation rate (120 mm/H, normal < 20 mm/H), increased serum total protein (93 g/L, normal 60–80 g/L), increased serum globulin (69.3 g/L, normal 20–30 g/L), decreased serum albumin (23 g/L, normal 35–50 g/L), increased serum cholinesterase (2860 U/L, normal 130–310 U/L). Index of liver and renal function test were all within normal ranges. Studies of coagulability were all within normal ranges. Rheumatism immunity combination test showed decreased IgA (4.69 g/L, normal 7.0–16.0 g/L), increased IgG (48.4 g/L, normal 0.7–4.0 g/L), suspicious positive ENA-AbSSA, weakly positive ENA-AbRo, and positive HEP2-ANA (1:100). PANCA, CANCA, dsDNA, and ACA-IgG were normal. Immunofixation electrophoresis test was normal. Light chain combination test showed increased serum light chain of LAM (6.22 g/L, normal 0.90–2.10 g/L), increased serum light chain of KAP (10.70 g/L, normal 1.70–3.70 g/L), increased urine light chain of LAM (13.5 g/L, normal 0.00–3.90 g/L), and increased urine light chain of KAP (57.1 g/L, normal 0.00–7.10 g/L). Serum tumor marker: NSE, CEA, cytokeratin 19 fragment, and CA125 values were all in normal ranges. Moreover, the antibody tests revealed negative for tuberculosis antibodies (IgM and IgG), negative for T-SPOT.TB, negative for human immunodeficiency virus, negative for EBV-CA IgG, negative for EBV-CA IgM, and negative for EBV-EA IgA, and that increased serum IgG4 (7.700 g/L, normal 0.020–2.000 g/L).

**Figure 1.**
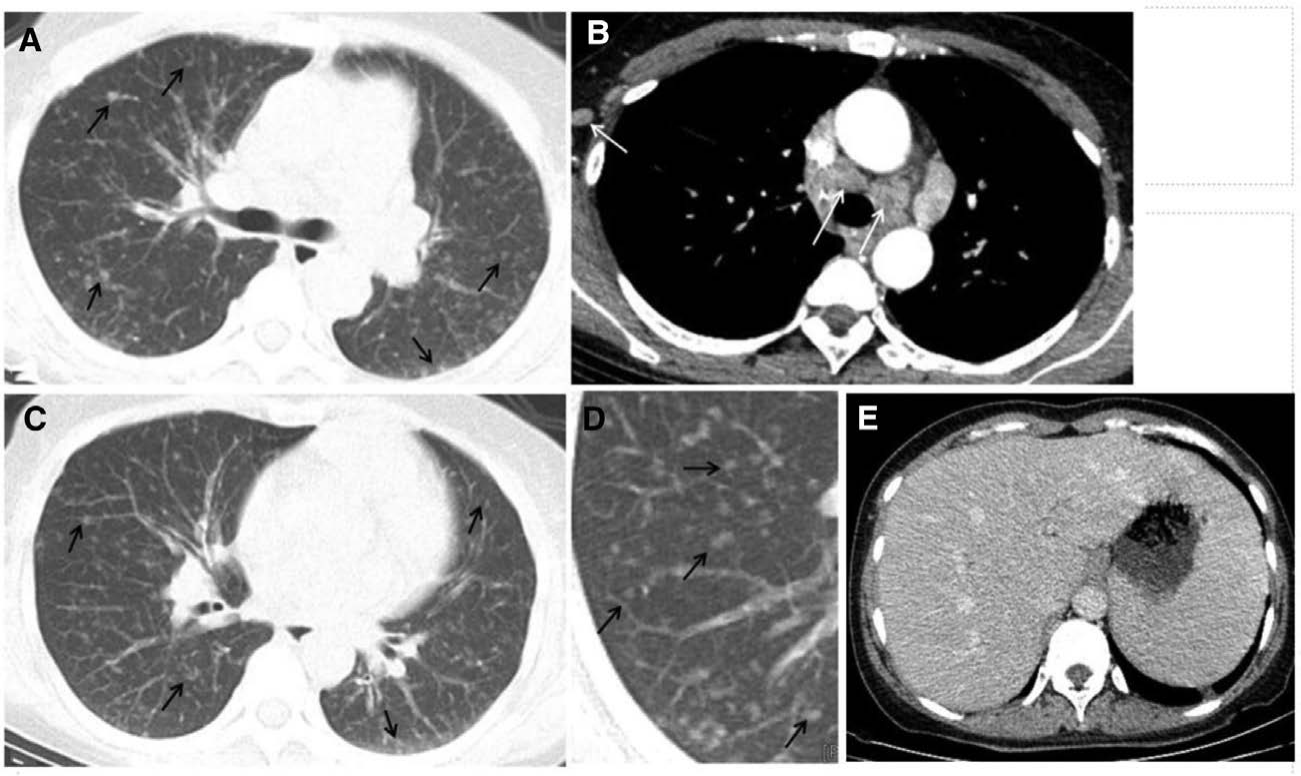
Axial contrast-enhanced chest CT scan: diffuse ground glass nodules in both lungs (A, C, D), multiple enlarged lymph nodes in mediastinum and axilla (B). Abdominal enhanced CT scan showed enlargement of the liver and spleen (E). CT = computed tomography.

Chest enhanced CT scan showed diffuse nodules of different sizes and densities in the bilateral lung fields, multiple enlarged lymph nodes in the mediastinum and bilateral axillas and no definite fusion changes among the lymph nodes (Fig. 1A–D). Abdominal enhanced CT scan showed enlargement of the liver and spleen, enlargement of abdominal aortic and inguinal lymph nodes (Fig. 1E). Pulmonary function tests revealed a mild restrictive defect with a reduced diffusing capacity. Color doppler ultrasound of the abdomen indicated enlargement of the liver and spleen.

**Figure 2.**
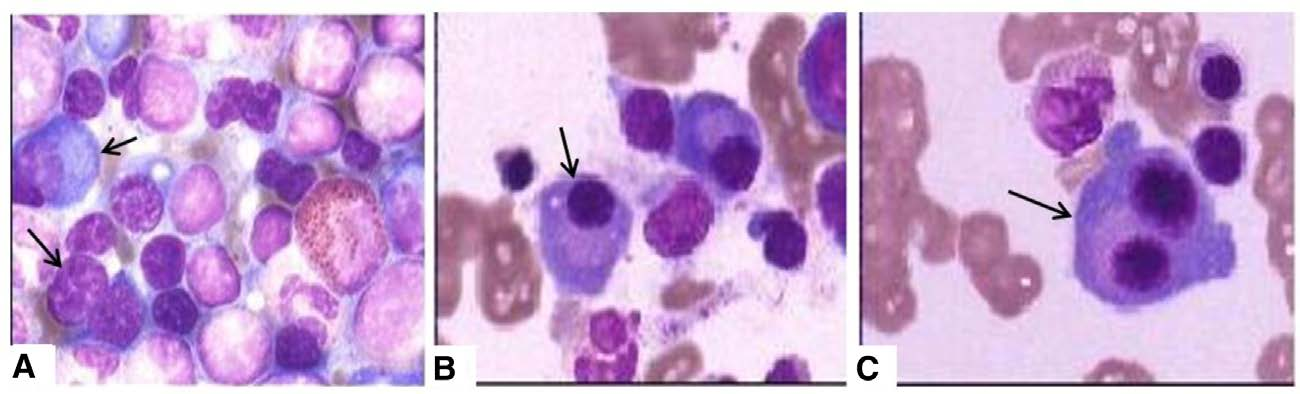
Bone marrow puncture + biopsy: hyperplasia was active in the granulocytes of the 3 lines (A), and abnormal plasma cells were classified by 9% (B, C), (H&E; original magnification, ×40).

Bronchoscopy revealed no endobronchial involvement. Subsequent biopsies of the mediastinal 4R group and 7th group lymph nodes by means of endobronchial ultrasound-guided transbronchial needle aspiration: lymphocytes and lymphoid tissues were observed. Furthermore, the enlarged inguinal lymph node was biopsied with needle aspiration, and showed reactive lymph node hyperplasia and large amount of plasma cell infiltration. Therefore, the patient was initially considered a plasma cell infiltration disease, especially lymphomas, and the hematological examination was further performed. Bone marrow biopsy showed hyperplasia was active in the granulocytes of the 3 lines and abnormal plasma cells account for 9% in all cells (Fig. 2).

**Figure 3.**
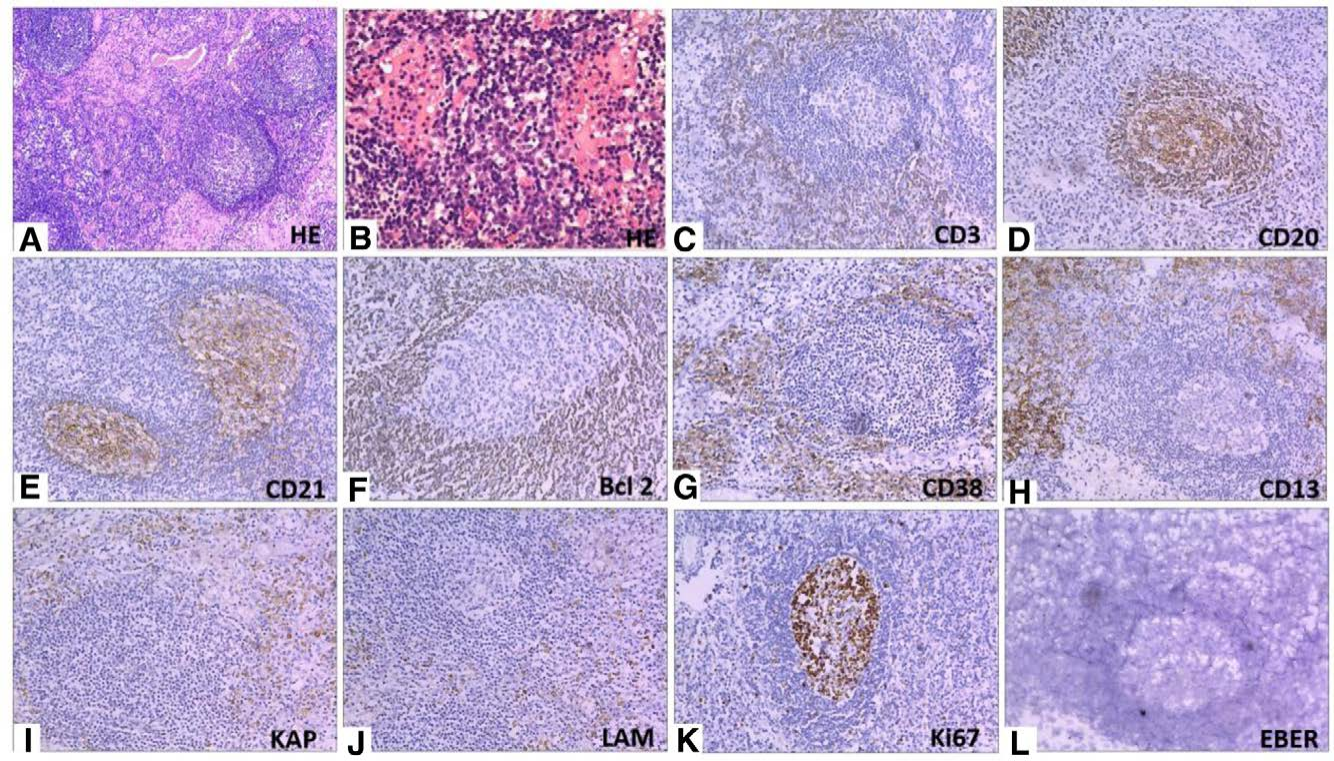
Histopathology. H&E staining (A, B), magnification × 100 and × 200, respectively (immunohistochemical staining (C–L), magnification × 200).

Subsequently, the patient underwent inguinal lymph node biopsy to obtain a more precise diagnosis. The enlarged right inguinal lymph node was resected completely, and hematoxylin and eosin staining of the biopsy samples showed lymphoid follicular hyperplasia, large amount of plasmacytoid cell infiltration seen in the follicle, combined with immunohistochemical examination results, consistent with reactive lymph node hyperplasia and large amount of plasma cell infiltration. Immunohistochemical showed CD3 (T lymphocytes+), CD20 (B lymphocytes++), Ki67 (lymphoid follicle germinal center+), Ckpan (‐), Bcl-2 (lymphoid follicle germinal center‐), EBER (‐), CD38 (plasma cells+), CD138 (plasma cells+), LAM light chain (+), KAP light chain (+), CD10 (‐), the Bcl-6 (lymphoid follicle germinal center+) (Fig. 3).

After consultation with a multidisciplinary team including pathologists, an oncologist, and a hematologist, diseases that require differential diagnosis such as sarcoidosis, tuberculosis metastatic tumor and other plasma cell disorders were ruled out, and MCD was finally diagnosed according to the pathology of inguinal lymph nodes biopsy and other clinical data.

The diagnosis was made that these findings were consistent with MCD, which mainly involved lymphatic system and presented with diffuse ground glass nodules in both lungs. Therefore, the patient was given prednisone acetate, 50 mg daily, and the symptoms of chest tightness, shortness of breath, cough and expectoration were significantly alleviated. Chest CT after 1 month of hormone therapy showed no significant changes than before. It suggests that hormone therapy alone is ineffective to CD. Then, the patient received systemic chemotherapy with cyclophosphamide, vincristine, and prednisone. After 6 cycles of chemotherapy, the patient chest CT and clinical symptoms improved.

## 3. Discussion and conclusions

MCD is divided into idiopathic MCD, human herpes virus 8-associated MCD, and polyneuropathy, organomegaly, endocrinopathy, monoclonal plasma cell disorder, skin changes-associated MCD.^[2]^ At present, the pathogenesis of CD is not completely clear, but interleukin, human herpes virus and HIV infection have been reported to play an important role in the pathogenesis of CD.^[4–6]^ This patient was not found to have any of the above risk factors. In addition, the patient had enlargement of the liver and spleen, but had no other symptoms of polyneuropathy, organomegaly, endocrinopathy, monoclonal plasma cell disorder, skin changes. Therefore, the patient was classified as idiopathic MCD.

Although CD can occur at any site, approximately 70% of CD occurred in the thorax, the mediastinum and hilum.^[7,8]^ Chest CT scan is of great value in the diagnosis and differential diagnosis of the disease. CT scan mainly present large soft tissue mass in lungs and multiple mediastinal lymph node enlargement. Multiple ground glass nodules with different sizes in both lungs are rare.

To date, Castleman disease remains incompletely understood due to its rareness and difficulties in clinical and pathological diagnosis. Diagnosis must depend on adequate immune typing.^[9]^ The IHC antigens were: Kappa/Lambda, CD20, CD3, CD5, CD138, human herpes virus 8 incubation-related nuclear antigen 1, etc. In this case, endobronchial ultrasound-guided transbronchial needle aspiration was used at the early stage, but no obvious abnormal pathological changes were detected. Lymph node follicular hyperplasia was confirmed after inguinal lymph node biopsy, and a large number of plasma cells infiltrated between lymph follicles. Therefore, lymph node resection or biopsy should be performed as far as possible for patients with suspected CD. According to the international consensus treatment guidelines for idiopathic MCD,^[10]^ this patient meets severe MCD, and the prognosis is poor.

In terms of the differential diagnosis of this patient, inguinal lymph node biopsy and bone marrow biopsy pathology indicated a large amount of plasma cell infiltration, and immunohistochemistry of inguinal lymph node tissue indicated that IgG4(+)/IgG(+) was 30%. Therefore, the differentiation with IgG4-related disease (IgG4-RD) was very important. IgG4-RD is a kind of immune mediated inflammatory disease, common clinical symptoms including sclerosing salivary gland inflammation, interstitial pneumonia, autoimmune pancreatitis, retroperitoneal fibrosis, sclerosing cholangitis, cholecystitis, etc.^[11,12]^ This patient had no clinical symptoms above. The pathological features of IgG4-RD include large amount of IgG4(+) lymph cells infiltration, occlusive phlebitis, etc.^[13,14]^ Pulmonary imaging finding of IgG4-RD is organized pneumonia or NSIP (nonspecific interstitial pneumonia). In this case, interleukin-6, erythrocyte sedimentation rate and C-reactive protein were significantly increased, and there was no increase of eosinophils in serum and alveolar lavage fluid. Besides, no striated fibrosis and occlusive phlebitis were found in lymph nodes and lung tissues, so the evidence supporting IgG4-RD was insufficient. In addition, glucocorticoid therapy is usually effective for IgG4-RD.^[11]^ However, after 1 month of treatment with prednisone acetate tablets, the patient clinical symptoms and chest CT were not improved. So the diagnosis of IgG4-RD for the patient was not supported further.

After 6 cycles of systemic chemotherapy with cyclophosphamide, vincristine, and prednisone, the patient chest CT and clinical symptoms improved. Currently, the patient is still receiving low-dose prednisone and cyclophosphamide orally for long-term maintenance treatment.

In conclusion, CD is a rare lymphoproliferative disease with varied clinical and imaging signs. CD that presents with diffuse parenchymal lung disease is extremely rare. It is easily confused with other diseases, identified diagnosis is depend on pathological examination. The accuracy of clinical subtype and histopathogenic type are important for treatment and outcome.

## Author contributions

**Conceptualization:** Xiaotong Guo.

**Data curation:** Xiaotong Guo.

**Investigation:** Fen Zhang.

**Project administration:** Kedong Zhang.

**Writing – original draft:** Xiaotong Guo.

**Writing – review & editing:** Caixia Zhu, Juan Chen, Kedong Zhang.
